# Palliative Care With Dementia and Family Caregiver Involvement (A Collaborative Symposium between the Hospice, Palliative, and End-of-Life Care and Nursing Care of Older Adults Interest Groups)

**DOI:** 10.1093/geroni/igaa057.2845

**Published:** 2020-12-16

**Authors:** Jenny van der Steen, Christopher Johnson, Sheryl Zimmerman

## Abstract

This collaborative symposium offered by the Hospice, Palliative, and End-of-Life Care and Nursing Care of Older Adults Interest Groups addresses palliative care including advance care planning considerations for family caregivers and persons with dementia. Family caregivers may need information about palliative and end-of-life care that is specific to the person, the situation or the stage of dementia. This symposium shows information needs also differ by country and setting. Conversations about symptoms, and about current and end-of-life treatment preferences need support from healthcare professionals. The symposium shows results of a study on video recordings with end-of-life preferences and how, as a stand-alone, they may not inform palliative care practice, and integration of information sources for advance care planning is needed. We will also show that a question prompt list with examples of questions to encourage family caregivers to ask healthcare professionals can and should have different contents for different countries as the content reflects socio-cultural differences. In more studies, participants clearly neede information on the disease trajectory and available services. Such needs go beyond need for information on pain and other symptoms, as family caregivers often appreciate opportunities for social activities for persons with dementia. A decision aid study shows that persons with dementia and family caregivers can participate in advance care planning conversations when supported by the right tools. We argue that local client participation is important when developing tools. Overall, the symposium highlights the need for tailored tools to support face-to-face conversations with all stakeholders to encourage person-centred caregiving.

